# A minimal model of elastic instabilities in biological filament bundles

**DOI:** 10.1098/rsif.2022.0287

**Published:** 2022-09-21

**Authors:** Chris Prior, Jack Panter, Halim Kusumaatmaja

**Affiliations:** ^1^ Department of Mathematical Sciences, Durham University, Durham DH1 3LE, UK; ^2^ Department of Physics, Durham University, Durham DH1 3LE, UK

**Keywords:** filament bundles, internal buckling, glaucoma, cell abcission

## Abstract

We present a model of a system of elastic fibres which exhibits complex, coupled, nonlinear deformations via a connecting elastic spring network. This model can capture physically observed deformations such as global buckling, pinching and internal collapse. We explore the transitions between these deformation modes numerically, using an energy minimization approach, highlighting how supported environments, or stiff outer sheath structures, favour internal structural collapse over global deformation. We then derive a novel analytic buckling criterion for the internal collapse of the system, a mode of structural collapse pertinent in many biological filament bundles such as the optic nerve bundle and microtubule bundles involved in cell abscission.

## 1. Introduction

Filamentary bundles (fibres embedded in an elastic matrix) are a common biological structure. There is ample experimental evidence showing how their elastic response is critical for their functionality. For example, collagen bundles are composed of linked tropocollagen molecules with a range of mechanical properties from compliant to rigid [1–3]. The optic nerve bundle is composed of rigid bundles of myelinated neurons embedded in connective tissues, and damage to this bundle because of pressure-induced buckling is considered a likely cause of glaucoma [4]. Similar nerve bundle structures play a crucial role in spinal cord injuries [5]. Microtubules are known to moderate their flexibility and alter their load bearing capacity in muscles [6,7].

There has been significant effort to reduce these diverse and complex elastic responses to generalizable elastic bundle models. Such models range from isolated filaments [7–9], to exact geometrical models [10–13] and multi-scale continuum approaches [14–17]. Their applications are diverse, including actin filaments [18], microtubules [19,20], collagen bundles [21], optic nerves [22,23], muscle fibres [14], fabric composites [24], carbon fibres [25] and mechanical ropes [26].

**Figure 1.**
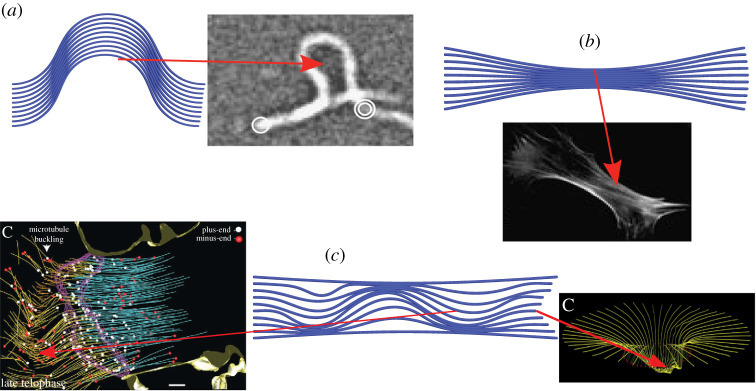
Three general classes of deformation modes: (*a*) a globally buckled state, here compared with the buckling of actin filaments [27] (adapted with permission, please be aware that this permission may not cover reuse under the OA agreement); (*b*) a pinched configuration, here compared with the basal organization of the actin filament network in an MEF fibroblast [28] (adapted with permission, please be aware that this permission may not cover reuse under the OA agreement); (*c*) internally buckled states, compared here with (left) microtubule bundle during cell abscission [29] (reproduced/adapted with permission which does not not cover reuse under the OA agreement) and (right) ‘cupping’ of the optic nerve in an eye that has suffered glaucoma [30] (adapted with permission, please be aware that this permission does not cover reuse under the OA agreement).

In this study, we develop a model that explicitly describes multiple filaments whose interactions are mediated through an elastic matrix, which we will show to be essential for capturing the surprising range of deformation modes exhibited by fibre bundles under compression. To our knowledge, there exists no fibre bundle model which has been demonstrated to be capable of describing these modes. In figure 1, we categorize these deformation modes into three types. Firstly, in figure 1*a*, the bundle may buckle globally, as observed for isolated actin and microtubule bundles [8,27]. Secondly, as shown in figure 1*b*, pinching (lateral contraction) may occur. This has been seen in the mouse embryonic fibroblast (MEF) [28], where the contraction of actin fibres was shown to be crucial in controlling nuclear shape. Finally, in figure 1*c*, buckling may instead occur internally, as is the case for microtubule bundles and the optic nerve bundle [4,29,31].

We are able to rationalize these deformation modes with three key parameters: the axial compression relative to the filament bending stiffness, the pinching load normalized against the interior filament bending stiffness and the non-dimensionalized ratio of the bending stiffness of the outer filaments to the inner filaments (representing, for example, the optic nerve bundle’s stiff outer sheath).

The principal feature of our model is the explicit coupling between filaments of fixed length via both Hookean springs and an overlap penalty. This combination of interaction forces is essential for two reasons. The first is so that the model captures physical deformations. The second is so that the buckling of one filament can potentially trigger further buckling, and a nonlinear cascade of response for the whole bundle. This coherent buckling is critical, in particular, for explaining the internal collapse mode.

The internal buckling mode is of particular interest here, in part because there appears to be no relevant buckling criteria existing in the literature, but mainly because of their functional role in biological bundles. As our model will confirm, the localized pile-up type behaviour of these deformations leads to significant spikes in local pressure. For the optic nerve, this could be crucial and deleterious, as external pressure can inhibit neuronal signalling action [32]. By contrast, for cell abscission this pressure is a desirable property as it locally weakens the bundle, providing a point for cleavage [29]. The pinching deformation modes are shown to lead to a more uniformly distributed internal pressure which is of a lower order of magnitude, in some sense making it a ‘safer’ deformation. It is thus important to know what factors will favour one mode over another. Overall, a key outcome of our analyses is to show the mechanical forces relevant for mediating the transitions between different bundle morphologies.

## 2. The model

### 2.1. Model geometry

**Figure 2.**
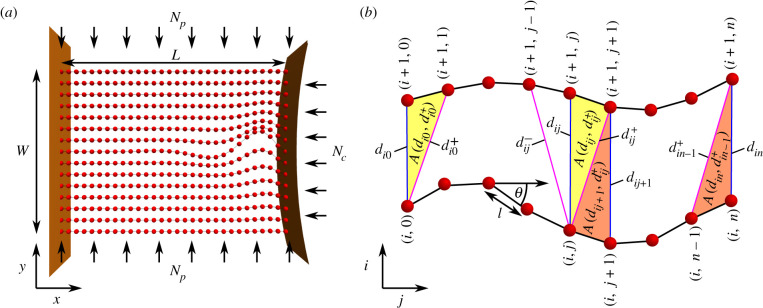
Model geometry. (*a*) The set of stacked filaments and the applied compressive (*N*_*c*_) and pinching (*N*_*p*_) loads. (*b*) Discretization of the filaments, where *l* is the segment arc length with angle *θ*, and with indices *i* labelling the filament number and *j* the arc length ordering along the filament. The spring distances *d* and triangular areas *A* are shown for both ends of the bundle and a representative middle section.

The basic model, illustrated in figure 2, comprises a set of *m* inextensible planar elastic rods **r**_*i*_, *i* ∈ 1, …*m*. Each rod, of the same arc length *L*, is represented as a discrete curve which is itself composed of *n* points (typically *n* = 200 here). Their Cartesian coordinates $(x_{ij},y_{ij}),j\in 1,\ldots n$, can be parametrized in terms of a set of angles *θ*_*ij*_

2.1 $$(x_{ij},y_{ij})=\sum_{k=1}^{\;j-1}\left(l\cos\theta_{ik},l\sin\theta_{ik}+\frac{iW}{m-1}\right),\;l=\frac{L}{n}.$$

The parameter *W* is the bundle’s width. Thus $\theta_{ij}=0,\;\forall i,j$ represents a set of straight filaments directed along the $\hat{x}$-axis: the bundle’s rest state. The *m* discrete rods have fixed positions at *j* = 0 for which *x*_*i*0_ = 0 and there is a vertical spacing *y*_(*i*+1)0_ − *y*_*i*0_ = *W*/(*m* − 1), as shown in figure 2*a*. The angles *θ*_*ij*_ determine the bending of the curves, as shown in figure 2*b*. In this work, the filaments are confined to two dimensions, as already a range of deformation modes are manifest, with their biological relevance highlighted in figure 1. More complex three-dimensional modes, such as torsional deformations, will be explored in future.

### 2.2. Energy functional

The set of filaments is then assigned the following energy functional:

2.2 $$E=E_{b}+E_{e}+E_{l}+E_{c},$$

where the total energy *E* is the sum of the bending energy *E*_*b*_, elastic interaction potential *E*_*e*_, loading potential *E*_*l*_ and geometric boundary constraint potentials *E*_*c*_. All parameters used to subsequently define the energy functional components are summarized in appendix A.

### 2.3. Bending energy *E*_*b*_

For each rod *i*, the bending energy takes the following form:

2.3 $$E_{b(i)}=\sum_{\;j=2}^{n}\frac{B_{ij}}{2l}\tan^{2}\left(\frac{\theta_{ij}-\theta_{i(j-1)}}{2}\right).$$

This curvature measure is based on the radius of a circumscribed circle encompassing adjacent edges of the curve, and is a standard discrete form [33]. In the limit *l* → 0, it becomes a finite difference approximation for the curvature. The coefficients *B*_*ij*_ represent the localized bending stiffnesses of the filament. Here, these values will be uniform along the filament length (*B*_*ij*_ = *B*_*i*_) and the only variation is between the outer bending stiffness *B*^*o*^ = *B*_1_ = *B*_*m*_ and inner bending stiffness *B*^in^ = *B*_*i*_, *i* = 2, 3…*m* − 1. Physically the ratio *B*_*r*_ = *B*^*o*^/*B*^in^ is used to model either surrounding support to the bundle or a stiff outer sheath, such as the epineurium and perineurium sheaths surrounding neuron fibre bundles [34].

### 2.4. Interaction energy *E*_*e*_

The interaction energy *E*_*e*_ in (2.2) is the sum

2.4 $$E_{e}=E_{e}^{1}+E_{e}^{1+}+E_{e}^{1-}+E_{e}^{2}.$$

The component $E_{e}^{1}$ is a set of Hookean spring potentials joining neighbouring filaments, dependent on the arc length paired distances *d*_*ij*_ between neighbouring springs (*i*, *i* + 1) at location *j* along their length (figure 2*b*). Formally, $E_{e}^{1}$ is the following Hookean potential:

2.5 $$E_{e}^{1}=\sum_{i=1}^{m-1}\sum_{\;j=0}^{n}lf\;(d_{ij}),$$

where *d*_*ij*_ = |***d***_*ij*_| , with the vector ***d***_*ij*_ expressed as

$$d_{ij}=(x_{(i+1)j}-x_{ij},y_{(i+1)j}-y_{ij}),$$

and the function

$$f\;(d_{ij})=\frac{K_{ij}}{2}{\left(\frac{d_{ij}-d_{0}}{d_{0}}\right)}^{2}.$$

Here *d*_0_ = 1/(*m* − 1) is the value of *d*_*ij*_ in the bundle’s rest configuration (all *θ*_*ij*_ = 0) and the constants *K*_*ij*_ are the spring stiffnesses. The model can allow *K*_*ij*_ to vary but in this study we choose a homogeneous connective tissue *K*_*ij*_ = *K*. The factor *l* in (2.5) makes *K* spring energy a density per unit length.

In addition, there are diagonal spring potentials $E_{e}^{1+/-}$ dependent on distances $d_{ij}^{\pm }$ between point pairs (*i*, *j*) and (*i*, *j* ± 1), as indicated in figure 2*b*. The first set of potentials, corresponding to the distances *d*_*ij*_, model the stretching of connective tissue linking the filaments, while the potentials associated with the diagonal distances $d_{ij}^{\pm }$ model the localized axial stretching of this tissue due to filament curvature. The diagonal spring potentials $E_{e}^{1\pm }$ are also Hookean potentials

2.6 $$E_{e}^{1\pm }=\sum_{i=1}^{m-1}\sum_{\;j=1}^{n}lf^{\pm }(d_{ij}^{\pm }),$$

where $d_{ij}^{+}=|d_{ij}^{+}|$ and $d_{ij}^{-}=|d_{ij}^{-}|$, with the vectors $d_{ij}^{+}$ and $d_{ij}^{-}$ expressed as

$$\begin{array}{rl} & d_{ij}^{+}=(x_{\left(i+1)(j+1\right)}-x_{ij},y_{\left(i+1)(j+1\right)}-y_{ij}),\\ & d_{ij}^{-}=(x_{\left(i+1)(j-1\right)}-x_{ij},y_{\left(i+1)(j-1\right)}-y_{ij}), \end{array}$$

and

2.7 $$f^{\pm }(d_{ij})=\frac{K_{ij}^{s\pm }}{2}{\left(\frac{d_{ij}^{\pm }-d_{0}^{\pm }}{d_{0}^{\pm }}\right)}^{2}.$$

Here $d_{0}^{\pm }=\sqrt{1/{\left(m-1\right)}^{2}+l^{2}}$ is the value of $d_{ij}^{\pm }$ in the bundle’s rest configuration. Note there is no $d_{i0}^{-}$ or $d_{\mathrm{in}}^{+}$ as these distances are formally set to zero.

The second interaction potential $E_{e}^{2}$ prevents the overlap of the filaments by penalizing each area $A(d_{ij},d_{ij}^{+})$ and $A(d_{i(j+1)},d_{ij}^{+})$, illustrated in figure 2*b*, if an area gets close to zero. $A(d_{ij},d_{ij}^{+})$ is defined by the triangle between the two vectors ***d***_*ij*_ and $d_{ij}^{+}$, such that $A(d_{ij},d_{ij}^{+})=|d_{ij}\times d_{ij}^{+}|/2$. Filament overlap is identified by $A(d_{ij},d_{ij}^{+})$ becoming zero, as for this to occur the length *d*_*ij*_ or $d_{ij}^{+}$ is zero, or the angle between ***d***_*ij*_ and $d_{ij}^{+}$ is *π*. For $A(d_{i(j+1)},d_{ij}^{+})$, similar reasoning applies, with $A(d_{i(j+1)},d_{ij}^{+})$ defined as $A(d_{i(j+1)},d_{ij}^{+})=|d_{i(j+1)}\times d_{ij}^{+}|/2$. The term *g*(*A*) we choose to penalize area *A* getting close to zero is

2.8 $$g(A)=C_{1}e^{-C_{2}A/A_{0}},$$

where *A*_0_ = *l*/2(*m* − 1) is the size of each triangle in the rest configuration. The parameter *C*_1_ is chosen to be *C*_1_ = 10^6^ in order to effectively prevent rod overlap. The parameter *C*_2_ determines the extent of compression required for the potential to become large. In most of the examples in this study, we use *C*_2_ = 100, so that *g*(*A*) is only significant if the area of a given triangle is reduced to less than 10% of its original size. This means the elastic matrix between the filaments acts like a linear material until the filaments come close to contact. A lower *C*_2_ value would mean the potential becomes larger for less significant compression (the value of the ratio *A*/*A*_0_) making the material act more like one with pronounced strain hardening. In §3.6, we briefly explore the consequences of lowering *C*_2_ and show this does not qualitatively alter our conclusions as to the nature of the system’s phase space. The specific form we choose for $E_{e}^{2}$ is

2.9 $$E_{e}^{2}=\sum_{i=1}^{m-1}\sum_{\;j=0}^{n-1}\left[g(A(d_{ij},d_{ij}^{+}))+g(A(d_{i(j+1)},d_{ij}^{+}))\right].$$

We experimented with a similar overlap energy penalty, in which a repulsive potential was applied if *d*_*ij*_ and $d_{ij}^{\pm }$ approached zero length. However, this was not as effective as the area penalty, as points on the filament could fit between two points on a neighbouring filament without the spring distances ever becoming small. A final point to make is that this energy does not prevent non-local self-overlap, occurring in globally buckled states when the load *N*_*c*_ ≫ 1 and the bending coefficient ratio *B*_*r*_ is of order unity. This can be remedied by adding an additional potential which penalizes non-local filament overlap, such as penalizing a point from approaching a line segment between two other points. However, such a term was found to significantly increase the computation time, and the range of forces used in this work mean that the local treatment was sufficient to prevent filament overlap. This allowed us to complete a significant range of parameter sweeps in a reasonable time frame.

### 2.5. Loading potentials *E*_*l*_

The loading energy *E*_*l*_ in (2.2) is the sum of two terms:

2.10 $$E_{l}=E_{l}^{1}+E_{l}^{2}.$$

The potential $E_{l}^{1}$ represents a compressive load $-N_{ci}\hat{x}$ applied to each rod *i* with the potential energy associated with the load taking the following form:

2.11 $$E_{l}^{1}=\sum_{i=1}^{m}N_{ci}x_{\mathrm{in}}.$$

In this work, we apply a uniform load *N*_*ci*_ = *N*_*c*_ for all *i*. The potential $E_{l}^{2}$ applies lateral compression force, perpendicular to the force *N*_*c*_ (along the *y*-direction). This is defined as

2.12 $$E_{l}^{2}=\sum_{i=1}^{m}\sum_{\;j=1}^{n}N_{\;p(ij)}(y_{ij}-y_{i0}).$$

In this study, we prescribe a uniform compressive force on the outer filaments, so that *N*_*p*(*ij*)_ = *N*_*p*_ if *i* = *m*, *N*_*p*(*ij*)_ = −*N*_*p*_ if *i* = 1 and *N*_*p*(*ij*)_ = 0 otherwise. We call this a pinching load *N*_*p*_ in what follows, as it produces pinched geometries. The *N*_*p*(*ij*)_ can also be readily chosen to model different systems. For example, pinching at only one end is relevant for optic nerve bundle shown in figure 1*c* where the scleral wall pinches the bundle. For other applications such as cell nuclei models, one could pinch at the middle to simulate the pinching of a nuclear cell.

### 2.6. Geometric boundary potentials *E*_*c*_

The geometric potentials impose boundary conditions such as pinned conditions (i.e. no end-to-end deflection), and clamped boundary conditions (i.e. no end-to-end deflection and fixed end tangents). The pinned case for which *y*_*i*0_ = *y*_in_ is

2.13 $$E_{c}^{1}=\sum_{i=1}^{m}H(y_{\mathrm{in}}-y_{i0}),$$

where H(0)=0,H(s)>0,∀s≠0. We use quadratic functions in the form *H*(*s*) = *C*_3_*s*^2^ with *C*_3_ a constant. Such a harmonic energy penalty is well established for imposing constraints for a wide range of systems [35,36]. In this study, a value of *C*_3_ = 10^5^ was used to ensure the conditions were strongly enforced. Clamped boundary conditions include the potential $E_{c}^{1}$ and an additional potential $E_{c}^{2}$ which constrains the filament ends to be parallel to the *x*-axis. It takes the form

2.14 $$E_{c}^{2}=\sum_{i=1}^{m}H(\theta_{i1})+\sum_{i=1}^{m}H(\theta_{\mathrm{in}}).\;$$

The same quadratic form and *C*_3_ value was used for this condition also.

With regard to the biological examples shown in figure 2, the pinned boundary conditions applied alone would be appropriate for the actin filament buckling (*a*), as buckling is induced by compressing the filaments between two microscope slides. Indeed the single filament model in the cited study [27] used pinned boundary conditions. By contrast, the optic nerve bundle (*c*) begins at the lamina cribrosa, which is an elastically stiff porous material through which the neuron fibres are threaded [4]. This implies the curvature would be limited in this region, so clamped boundary conditions are appropriate. In addition, the pinching of the scleral wall restricts end movement of the filaments, so the addition of pinned conditions would also be appropriate. Similarly in the cell abscission example [29], there is an additional stiff elastic matrix structure (the mid-body structure) which deforms during abscission, which would also probably restrict curvature. We use both pinned and pinned and clamped conditions in what follows.

### 2.7. Non-dimensionalization

We non-dimensionlize the system parameters with a reference length corresponding to the width of the bundle *W*, and a reference bending stiffness *B*_*s*_ = 0.01. This yields non-dimensionalized parameters *L*′ = *L*/*W*, *N*_*c*_′ = *N*_*c*_/(*B*_*s*_*W*^2^), *N*_*p*_′ = *N*_*p*_/(*B*_*s*_*W*^2^) and *K*′ = *K*/(*B*_*s*_*W*^2^). The length scaling is chosen to ensure the parameter *m* determines the density of packing of the filaments through the rest separation *d*_0_ = 1/(*m* + 1). Hereafter, we drop the primes and all quoted parameters are assumed non-dimensionalized unless otherwise stated.

### 2.8. Locating energy minima

To find stable states, *E* in equation (2.2) was minimized using the L-BFGS algorithm [37,38], a memory and computationally efficient minimization algorithm that we have shown previously to be suitable for high-dimensional buckling models [39,40]. The explicit form of the gradients ∂*E*/∂*θ*_*ij*_ used to construct the gradient and approximate Hessian matrix are given in appendix B.

## 3. The phase space

### 3.1. External and material parameters

The triplet (*N*_*c*_, *N*_*p*_, *B*_*r*_) forms a set of externally acting parameters. This is clear for the loads *N*_*c*_, *N*_*p*_. The ratio *B*_*r*_ is classed as external as it is used to model either a surrounding support to the bundle or a stiff outer sheath as discussed above. The parameters *K* (connective medium spring stiffness), number of rods *m*, filament length *L* and internal filament bending rigidity *B*^in^ are the material parameters.

In this work, the set (*N*_*c*_, *N*_*p*_, *B*_*r*_) will parametrize the phase space of configurations and we find that they govern the transitions between the differing bundle geometries which minimize the system’s energy. We will then see how the material parameter set (*K*, *m*, *B*^in^, *L*) alter the nature of this phase space, in particular how they determine the critical values of these parameters at which the transitions occur.

#### 3.1.1. Deformation classification

**Figure 3.**
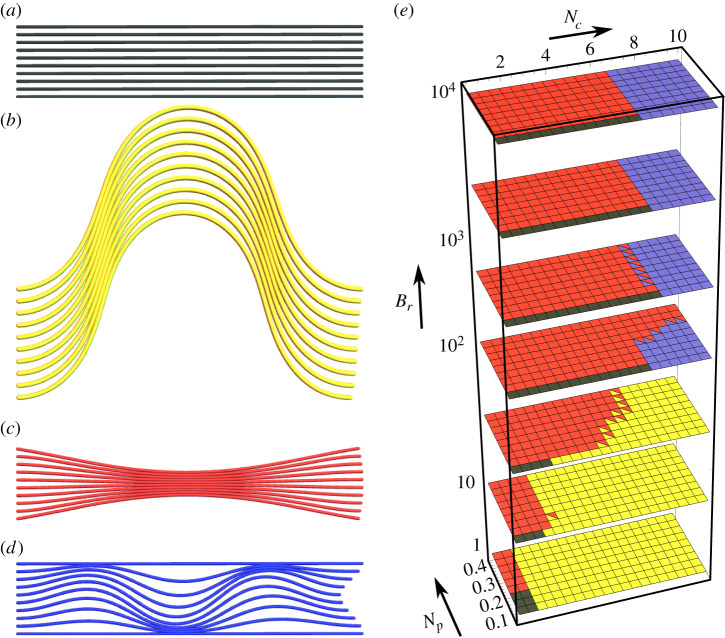
Model energy minima and their distribution in phase space. (*a*–*d*) Exemplars of the four energy minima of the model, showing the undeformed, globally buckled, pinched and internally buckled structures, respectively. (*e*) A representative phase space of the model’s energy minima for parameters detailed in the text. The colours match the colours in (*a*–*d*). Each square represents a parameter triplet (*N*_*c*_, *N*_*p*_, *B*_*r*_) and squares with two colours indicate those minimum types have energies within 1% of each other.

Overall, four stable geometry classes are found, which are visualized in figure 3. These classes are the unbuckled system shown in figure 3*a*, and the three deformed systems (globally buckled, pinched and internally buckled), shown in figure 3*b*–*d*, respectively. We highlight the fact the globally and internally buckled configurations are ‘symmetrically’ buckled, in the sense they buckle with the same mode and direction. By contrast, in the pinched configurations, the upper and lower filaments buckle in opposing directions. The configurations shown in figure 3 are archetypal minima of the system, which occur either in the absence of pinching (globally and internally buckled configurations) or the compressive load (asymmetrically pinched case). In general, when both loads are present, we use these archetypal configurations to define three characteristics of each geometry, whose relative degree is used to categorize the configurations. They are:

(i) *B*_*r*_〈|*κ*|〉: the mean absolute moment, an average of the curvature |tan^2^((*θ*_*ij*_ − *θ*_*i*(*j*−1)_)/2)| along the filament length, then averaged over all filaments. We multiply by *B*_*r*_ to account for the fact deformation is less for a given load in the outer filament.
(ii) *B*_*r*_〈|*κ*|〉^*o*^ and *B*_*r*_〈|*κ*|〉^*i*^, the quantity in (i) restricted, respectively, to the outer filaments and inner filaments only.
(iii) *P*: the pinching parameter. The pinched configurations have filaments which buckle in the opposite directions either side of the centre, whilst for the internal/global buckling the filaments buckle in the same direction. To measure the degree of pinching, we split the rods into the upper half *i* > (*m* + 1)/2 and the lower half *i* < (*m* + 1)/2 (or split around *m*/2 if *m* is odd). Then we calculate the weighted difference in angle between paired filaments from the upper and lower rods:
  3.1 $$P=\frac{1}{n}\sum_{\;j=1}^{n}\sum_{i=1}^{m_{h}}\frac{\left|\theta_{ij}-\theta_{m-1j}\right|}{\left|\theta_{ij}|+|\theta_{m-1j}\right|}.$$
  *m*_*h*_ = *m*/2 if *m* is even and *m*_*h*_ = (*m* − 1)/2 if *m* is odd. The average of this over all *j* and *i* is a number between 0 and 1, *A* > 0.75 is classed as dominantly asymmetric.

The mean absolute moment *B*_*r*_〈|*κ*|〉 is simply used to discriminate unbuckled and buckled configurations. The globally buckled configurations have particularly large values of this parameter. If *B*_*r*_〈|*κ*|〉 is greater than 0.00001 then the configuration is classed as buckled. We must then identify what type of buckling is present.

A large value of the ratio *B*_*r*_〈|*κ*|〉^*i*^/*B*_*r*_〈|*κ*|〉^*o*^ indicates structures with significant internal buckling characteristics (we use a ratio greater than 5). Otherwise, we must establish whether the structure is (globally) buckled or pinched, which is done using the pinching parameter *P*.

**Figure 4.**
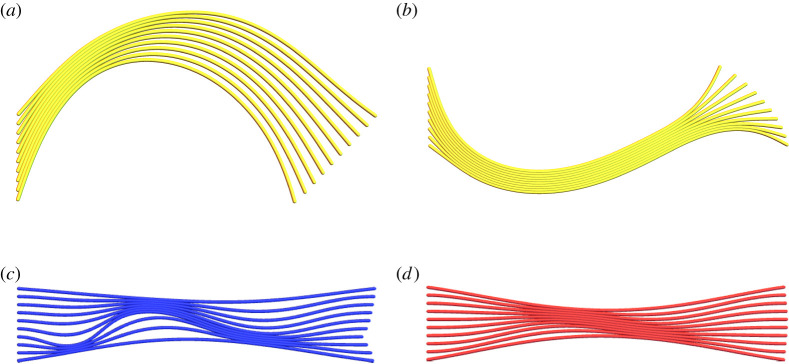
Examples of model energy minimum configurations with significant compressive and pinching loads present. Panel (*a*) is a globally buckled configuration with *N*_*c*_ = 0.5, *L* = 5, *B*_*r*_ = 1, *N*_*p*_ = 0.05. Panel (*b*) has a more significant pinching load *N*_*p*_ = 0.3. Both (*a*) and (*b*) are classed as globally buckled. Panel (*c*) is a configuration with *N*_*c*_ = 9, *L* = 5, *B*_*r*_ = 100, *N*_*p*_ = 0.3 which has mixed pinched internal buckling characteristics. This is classified as internally buckled in our system. Panel (*d*) is a configuration with *N*_*c*_ = 9, *L* = 5, *B*_*r*_ = 100, *N*_*p*_ = 0.35 which has mixed pinched-internal buckling characteristics. This is classified as asymmetrically pinched.

Generally we find the pinching parameter *P* is either very small or very close to 1, with small *P* indicative of systems with highly symmetric deformations such as global or internal buckling. We do find examples where there is a competition in classification due to the presence of both symmetric and asymmetric deformation modes. For example, globally buckled configurations with significant pinching forces are shown in figure 4*a*,*b*. The pinching load clearly affects the configuration, compared with figure 3*b*, but the dominant global characteristic is clear. Similarly in figure 4*c*, we see a configuration which is internally buckled but has some significant pinching. With a slight increase in pinching, figure 4*c* transforms to figure 4*d* and becomes classed as asymmetrically pinched. Note that significantly mixed pinched/internally buckled behaviour is only observed for mid-range *B*_*r*_ values (we consider [1, 10 000]) in our study. For higher *B*_*r*_ (say greater than 500) the symmetric and internally buckled configurations are significantly pronounced.

### 3.2. Surveying the phase space of deformation

We survey the available deformation modes over the parameter ranges *N*_*c*_ ∈ [0.5, 10], *N*_*p*_ ∈ [0, 0.4] and *B*_*r*_ ∈ [1, 10 000] for the phase diagram in figure 3*e*. In figure 3*e*, we have also used *L* = 5, *K* = 2, *m* = 10, *B* = 1. This range incorporates biologically important example bundles such as the optic nerve, as we will demonstrate in §4. The spring constant *K* is non-dimensionalized with respect to the bending coefficient. This value has the same order of magnitude as the bending stiffness so that both effects are important to the system’s behaviour (we vary this value shortly). We initialize the energy minimization in each of the four archetypal states, and then locate the local energy minima. When there are two local minima with energies within 1% of each other, these are marked with two colours.

We now explore the stability ranges and transitions in the phase diagram. We begin at (*N*_*c*_, *N*_*p*_, *B*_*r*_) = (0, 0, 1) in the undeformed geometry. This is a bundle with no external protective support (*B*_*r*_ = 1). As the pinching load *N*_*p*_ is increased, the magnitude of the pinching deformation shown in figure 3*c* simply increases smoothly. There is no apparent instability. By contrast, if *N*_*c*_ is increased instead, a sudden transition to the globally buckled state occurs, shown in figure 3*b*, governed by the classical Euler buckling criterion. Next, upon increasing the external support parameter *B*_*r*_, the globally buckled configuration is suppressed due to the extreme energetic penalty associated with deforming the stiff outer rods. At large *B*_*r*_ > 100, as *N*_*c*_ is increased, both the undeformed and pinched states suddenly collapse to an internally buckled state, such as that shown in figure 3*d*, at some critical value of *N*_*c*_, denoted by $N_{c}^{*}$.

There is a tendency for domination of the pinching configurations when *N*_*p*_ = 0 at lower *N*_*c*_. This is not a surprise, as the continuum version of elastic filament models does not have a straight equilibrium *θ* = 0 in the presence of a lateral force (see [41] eqn (99)). So the presence of a load *N*_*p*_ should always lead to some deformation. As discussed above, we find the degree of pinching to grow steadily with *N*_*p*_ (the example shown in figure 3*c* is one of the more exaggerated examples). For the high *B*_*r*_ cases, the degree of pinching is severely restricted.

### 3.3. Additional examples

We focus on the same phase space, the parameter ranges *N*_*c*_ ∈ [0.5, 10], *N*_*p*_ ∈ [0, 0.4], *B*_*r*_ ∈ [1, 10 000], and consider phase space plots for various boundary condition types, number of filaments *m* and spring stiffness *K*.

#### 3.3.1. Pinned boundary conditions

**Figure 5.**
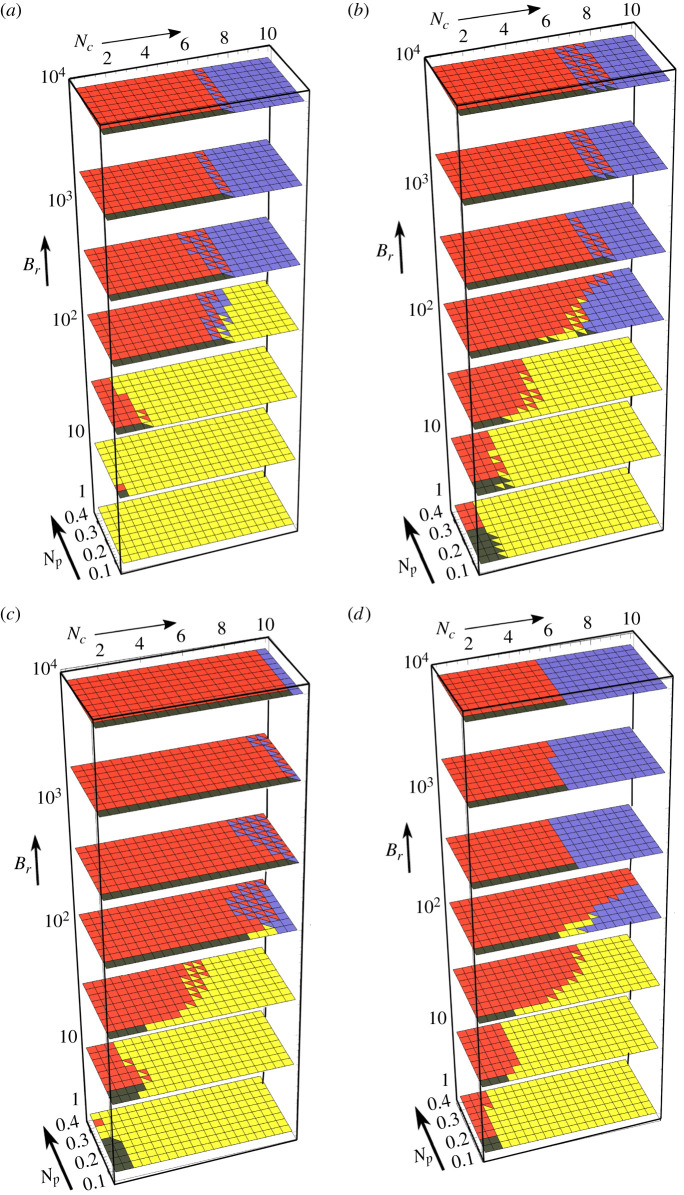
Illustrations of the phase space of the elastic filament model based on the parameters *N*_*c*_, *N*_*p*_, *B*_*r*_. Panel (*a*) shows results for 10 filaments with a length/width ratio of 5 and pinned boundary conditions. Panel (*b*) shows results for 20 filaments with a length/width ratio of 5 and clamped boundary conditions. Panels (*c*) and (*d*), respectively, have a spring stiffness twice and half of the results in figure 3*e*.

The minimum energy configurations for *m* = 10, *L* = 5, *K* = 2, *B* = 1 and only the pinning constraint energy $E_{c}^{1}$ are shown in figure 5*a*. This differs from the initial case which also has clamped constraints. For *B*_*r*_ = 1, the globally buckled configuration is the only configuration present. This contrasts with the clamped case shown in figure 3*e*, where the Euler buckling criteria ensure that global buckling is suppressed until the Euler criterion *N*_*c*_ > 4*π*^2^/25 is satisfied (as *B* = 1). For pinned boundary conditions, the critical criteria is *π*^2^/25 ≈ 0.395 which is always met within our *N*_*c*_ range. A second contrasting feature to the clamped results is that, for *B*_*r*_ = 1–33 (the first three layers of the plot), the globally buckled configuration dominates for lower *N*_*c*_. If we compare, for example, the results for *B*_*r*_ = 33, there is little pinching above *N*_*c*_ > 3, while pinching is observed up to loads of *N*_*c*_ = 7 in the clamped case. At *B*_*r*_ = 100 (fourth layer up) we also note that, in the pinned case, there are globally buckled configurations for high *N*_*c*_, whereas there are no such globally buckled minima in the clamped case. Hence the extra boundary constraint is important in this regime. By contrast, the results are largely very similar for the higher *B*_*r*_ regime, indicating the boundary conditions are not so fundamental for the globally buckled deformations.

#### 3.3.2. Twenty rods

The minimum energy configurations for *m* = 20, *L* = 5, *K* = 2 and *B* = 1 with both pinned and clamped constraints are shown in figure 5*b*. These differ from the results shown in figure 3*e* only by the number of rods. There is significant similarity between the two results so there are only a couple of features to highlight. First, we note that for intermediate values of *B*_*r*_ the pinching configurations disappear for lower *N*_*c*_, indicating a slight preference towards buckling when the outer support is not too strong. Second, for higher *B*_*r*_, while the *N*_*c*_ values at which internal buckling becomes a minimal energy configuration are the same, there is a range of *N*_*c*_ values over which both the pinching and global buckling configurations are effectively bistable. This is not present in the *m* = 10 case.

#### 3.3.3. Varying matrix (spring) stiffness

The minimum energy configurations for *m* = 10, *L* = 5, *K* = 4 and *B* = 1 with both pinned and clamped constraints are shown in figure 5*c*. This has twice the spring stiffness of the results shown in figure 3*e*. The preferred configurations for low *B*_*r*_ are nearly identical in both cases and the only significant difference manifests for high *B*_*r*_, where the critical *N*_*c*_ value at which internal buckling becomes an energy minimum is significantly increased. The variation in the curvature of the filaments inherent to this mode will naturally lead to significant stretching of the spring matrix, which explains why the parameter *K* has a significant effect on the critical internal buckling condition. Further evidence of this is seen in figure 5*d*, which corresponds to the minimum energy configurations for *m* = 10, *L* = 5, *K* = 1 with both pinned and clamped constraints. The only significant difference is the lower *N*_*c*_ value at which internal buckling occurs for high *B*_*r*_.

### 3.4. Forces in the system

**Figure 6.**
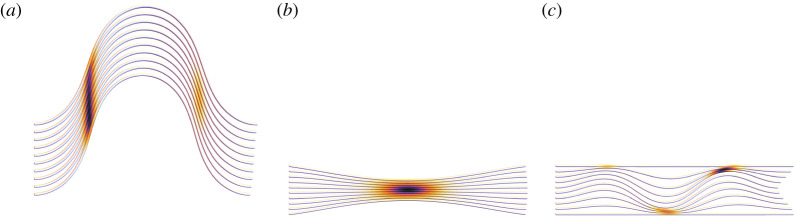
Internal interaction pressures of various states. The darker colours represent higher pressures. Panel (*a*) the globally buckled state, panels (*b*) and (*c*) show these distributions for the pinched and internally buckled configurations, respectively.

One final aspect of these minima which we investigate is the internal pressure caused by the spring forces, which can be computed as the partial derivatives of the interaction energy *E*_*e*_ with respect to the coordinates (*x*_*i*_, *y*_*i*_). The magnitude of their distributions are shown in figure 6*a*–*c*. There are clear spikes where the filaments are pressed together. The peak pressure was found to be an order of magnitude higher for the buckled configurations compared with the pinched case, indicating such geometries would be particularly prone to localized damage for individual filaments. As discussed in the Introduction, this can be either a positive or negative feature of the bundle’s mechanical response. The more uniform deformation of the pinch leads to more benign interactions.

### 3.5. Biological relevance

To further illustrate the potential biological relevance of these results, we argue that the material parameters we consider are relevant for the optic nerve. The optic nerve has a width and length of 3.55 and 28 mm, respectively [42], leading to the scaled length of *L* = 7.88 in our model. Young’s modulus for the outer sheath has been estimated to be *E*_*o*_ ≈ 44.6 MPa and the optic nerve itself *E*_*i*_ ≈ 5.4 MPa, respectively [42]. These values can be translated to filament bending coefficients via *πER*^4^/2, assuming a circular cross section with radius *R*. For the outer sheath, *R*_*o*_ = 0.37 mm [42]. The optic nerve thickness is typically around 3.55 mm, covering about 15 neuron sub-bundles, which leads to a nerve’s width of *R*_*i*_ = 3.55/30 = 0.12 mm. Thus, *B*_*r*_ = *B*^*o*^/*B*^in^ ≈ 10^4^. Finally, the typical inter-ocular pressures are 6.75 − 33.75 × 10^3^ MPa, which gives a range of *N* ∈ [6, 35] in our model. These parameters put the bundle in the regime where internal buckling dominates, suggesting optic nerve buckling in glaucoma can occur depending on the density of the collagen matrix which fills the space between the optic nerve bundles.

### 3.6. Elastic matrix model extensions

**Figure 7.**
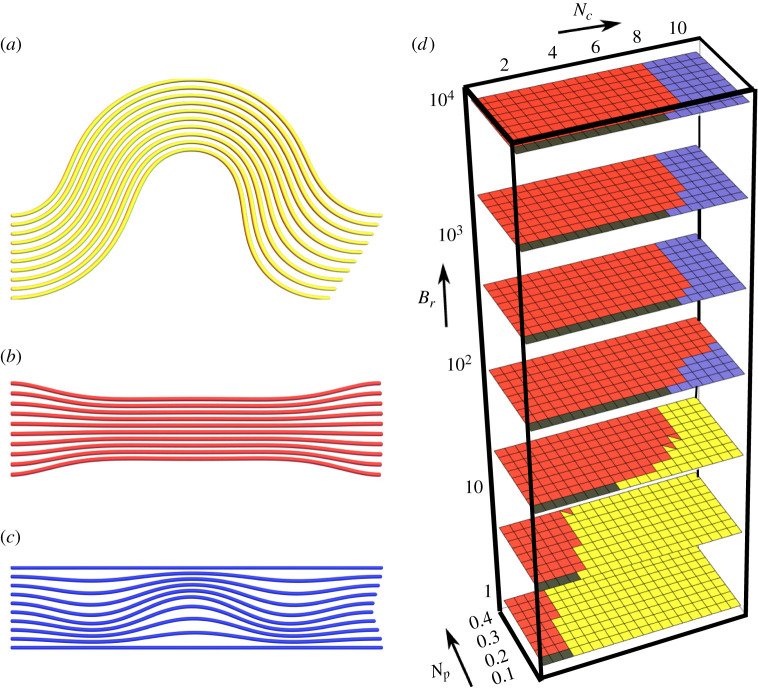
Model energy minima and their distribution in phase space for a strain hardening matrix (*C*_2_ = 40 in the interaction potential $E_{e}^{2}$). The parameter set is otherwise the same as for figure 3. Panels (*a*–*c*) are exemplars of the three deformed energy minima of the model present for comparison with those in figure 3. Panel (*d*) is the representative phase space of the model’s energy minima for parameters detailed in the text.

As discussed in §2.4, lowering the value of *C*_2_ makes the inter-fibre matrix act more like a strain hardening material. To explore the influence of this, we compile energy minima for the same parameter set as in figure 3*e*, but with the lower *C*_2_ value of *C*_2_ = 40. This means the potential $E_{c}^{2}$ becomes large when the matrix material is compressed below approximately 30% of its initial area. The results shown in figure 7 show only modest differences in comparison with figure 3*e*: at *B*_*r*_ = 100 there is an increase in the dominance of pinching, and the force required for internal buckling increases slightly. The similarity between all phase diagrams presented throughout suggest that our qualitative conclusions generalize across different elastic matrix models. Furthermore, the energy approach to our model means that is straightforward to replace the Hookean potential in equation (2.5) with more complex models of biological elasticity, if specific systems are to be investigated in the future.

## 4. Internal buckling

As discussed in the Introduction, the internal buckling transition is of particular biological significance. An important example of this is shown in figure 1*c*, in which asymmetric cupping of the optic nerve is observed in glaucoma sufferers. This is not readily explained by linear elastic models [22,43]. The extreme localized pressures in the internally buckled state might explain the damage caused to single elements of the whole bundle, thus leading to localized sight loss, the defining property of glaucoma's insult.

### 4.1. The internal buckling criterion

**Figure 8.**
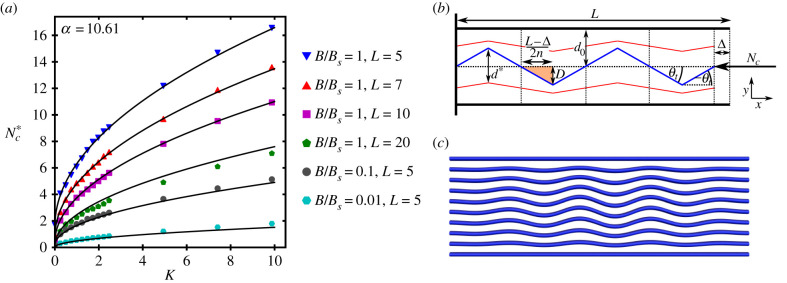
The model for the internal buckling criteria. (*a*) The variation of the critical buckling loads $N_{c}^{*}$ for internal buckling from the undeformed state, with spring stiffness *K*. A variety of filament lengths *L* and bending stiffnesses *B* are shown. Points represent simulation data, solid lines the model shown in equation (4.1). The fitting parameter is *α* = 10.61. Panels (*b*) and (*c*) depict the set-up and justification of the internal buckling model. Panel (*b*) shows a flexible, inextensible filament (blue) of length *L* buckles into *n* triangular kinks of amplitude *D*, under a load *N*_*c*_ displacing the end of the filament a distance Δ. The outer filaments shown in black are rigid. The red curve represents a neighbouring filament in a general bundle, its amplitude of buckling is smaller than the (assumed) central blue rod. The separation d* which determines the stretching is shown, as is the relaxed separation *d*_0_. Panel (*c*): in the internal buckling mode, the distortion peaks at the central filament and decays laterally while the wavemode remains stable.

As observed in the previous section, the model suggests the critical load at which this internal deformation occurs is consistent for a sufficiently strong external support parameter *B*_*r*_ > 100, independent of the pinching force *N*_*p*_, and varies with the model parameters. This suggests a clear buckling criterion could be developed for such systems which depends on the material parameters *K*, *B*^in^, *L*, *m*. To characterize this criterion, we chart the critical load $N_{c}^{*}$ at which the straight configuration collapses into the internally buckled state in figure 8*a*, for a variety of spring stiffnesses *K*, filament lengths *L*, and bending stiffnesses *B*. We fix *m* = 10, *N*_*p*_ = 0 and *B*_*r*_ = 10 000. The solid lines represent the following analytic prediction for the critical load $N_{c}^{*}$:

4.1 $$N_{c}^{*}=\alpha \sqrt{\frac{KB^{\mathrm{in}}}{L}}+\frac{n_{e}^{2}\pi^{2}B^{\mathrm{in}}}{L^{2}},$$

where *n*_*e*_ is minimum Euler buckling mode, *n*_*e*_ = 1 for pinned boundary conditions and *n*_*e*_ = 2 for clamped conditions, and *α* = 10.61 (we will shortly discuss the relevance of this value). Given the complexity of the model and the deformations involved, this is a remarkably simple formula and, as indicated in figure 8*a*, it is also highly accurate. We now discuss its derivation in some detail as it provides significant insight into the nature of the instability and the factors which determine it.

### 4.2. A discrete model

We make an energy estimation of the internally buckled state by assuming a triangular shape for the buckled filaments, as indicated in figure 8*b*, with the amplitude of buckling decaying laterally to zero, as in the numerically obtained minima shown in figure 8*c*. The indicated angle *θ*_*t*_ dictates both the buckling frequency and, through the fixed length criteria, the amplitude of buckling *D*. Both are functions of the distance Δ through which the force *N*_*c*_ does work. This allows us to explicitly calculate the energy in equation (2.2) and minimize it via a two-step process: first we find the load at which the system becomes unstable when Δ = 0, then we minimize the energy at this load for the wavemode *n* at which this occurs. We begin by only assuming *m* = 3 so that only the middle filament actually deforms. The general *m* formula is then a straightforward extension of this.

With reference to the undeformed state, the energy of the simple internally buckled state is

4.2 $$E_{int}=E_{l}+E_{e}+E_{b},\;E_{l}=-N_{e}\Delta .$$

Here *E*_*l*_ is the work done by the load *N*_*e*_ bending the middle filament. All parameters used to define the components of *E*_int_ are summarized in appendix A. Note that in this simplified model we are not including the boundary constraints. It is clear in the full nonlinear solutions that there is a decay in amplitude towards the end of the filaments, see figure 8*c*, owing to the need to satisfy the boundary conditions. So we are assuming this load *N*_*e*_ is the *excess* load over the Euler result.

### 4.3. The interaction energy *E*_*e*_

With three rods, *d*_0_ = 1/(*m* − 1) = 1/2, and the total spring energy is:

4.3 $$E_{e}=12n\;\int_{0}^{(L-\Delta )/2n}\frac{K}{2}\frac{{\left(d(x)-d_{0}\right)}^{2}}{Ld_{0}^{2}}\;dx.$$

Here, the integral is the elastic deformation energy associated with the shaded triangle shown in figure 3*a*, which takes into account both the energy of compressing the springs below the triangle, and extending the springs above. The factor of 1/*L* is used to ensure that as we vary *L*, there are the same number of springs per unit length to match our simulation set-up. The prefactor of *n* accounts for the total deformation energy of the 2*n* copies of the shaded triangle and the fact that there are six spring connections for each point in our model (four diagonal, two direct, so that in our earlier notation this is the sum $E_{e}^{1}+E_{e}^{1+}+E_{e}^{1-}$). We deduce

4.4 $$d(x)=x\frac{2nD}{L-\Delta }+d_{0},\;\text{where}\;D=\sqrt{\frac{\Delta }{4n^{2}}(2L-\Delta )},$$

and we can therefore evaluate equation (4.3) to give

4.5 $$E_{e}=\frac{K}{Ln^{2}}\Delta (L-\Delta )(2L-\Delta ).$$

Finally we note that, as we seek to derive an instability criterion, we can assume the overlap potential would be negligible and hence equation (4.5) represents the total interaction energy of the system.

### 4.4. The energy *E*_*b*_

To derive *E*_*b*_, we take a similar approach as in the derivation of the discretized simulation bending energy. The acute angle *θ*_*t*_ of the shaded triangle is

4.6 $$\theta_{t}=\arctan\left(\frac{2nD}{L-\Delta }\right).$$

We approximate the curvature energy as the difference between the two angles of the triangle as marked in figure 8*a*. Using the arctan formula of our model, we define the curvature *κ*_*t*_ of each triangle as

4.7 $$\kappa_{t}=\frac{1}{l_{t}}\tan^{2}\left(2\arctan\left(\frac{D}{l_{t}}\right)\right),\;l_{t}=\frac{L-\Delta }{2n}.$$

Thus the curvature energy, averaged over the 2*n* triangles is

4.8 $$\begin{array}{rl} E_{b} & =\frac{2nB^{\mathrm{in}}}{2l_{t}}\tan^{2}\left(2\arctan\left(\frac{D}{l_{t}}\right)\right)\\ & =\frac{2n^{2}B^{\mathrm{in}}}{L-\Delta }\tan^{2}\left(2\arctan\left(\frac{2nD}{L-\Delta }\right)\right). \end{array}$$

### 4.5. Instability as a balance between bending and matrix stretching

We take the derivative of equation (4.2) with respect to Δ (using equations (4.5) and (4.8)) and find the critical excess force *N*_*e*_ when Δ = 0 to be

4.9 $$N_{e}=\frac{KL}{n^{2}}+\frac{16n^{2}B^{\mathrm{in}}}{L^{2}}.$$

The first term represents the energy due to spring stretching. It decreases with *n* as the fixed length of the filaments means higher mode deformations have lower amplitude and hence less stretching of the elastic matrix joining them. The second term represents the bending energy which increases with *n*. Thus, we see the minimum of this function will be intermediate in *n*. An optimal *n*, denoted by n*, is found by minimizing *N*_*e*_ with respect to *n*. Substitution of this lowest-load mode n* into equation (4.1) returns the critical buckling load

4.10 $$N_{e}=8\sqrt{\frac{KB^{\mathrm{in}}}{L}}\;\mathrm{and}\;n^{*}=\frac{K^{1/4}L^{3/4}}{2{\left(B^{\mathrm{in}}\right)}^{1/4}}.$$

This also predicts (approximately) the expected wavemode of the buckling, which is increased by both length and matrix stiffness. Reflecting on this, the coefficient 8 is entirely determined by our approximation of the shape of the buckled configuration as a series of triangular kinks, and so cannot be expected to accurately represent the true minimum energy buckled configuration. It is reasonable therefore to treat the pre-factor as a fitting parameter *α*. As mentioned above, we add on the critical Euler load which accounts for the boundary conditions to give equation (4.1).

### 4.6. Accounting for neighbouring deformations

To account for the decay in buckling amplitude from the central of the bundle when *m* > 3 (seen in figure 8*c*), we assume neighbouring rods are also buckled, as in the red curves shown in figure 8*b*. This leads to a lower effective stretching d* which will be less than the value *d* used in the three-rod case described above. We should expect the variation in neighbouring rod curvature to vary as a function of the undeformed separation 1/(*m* − 1), the equilibrium separation of the rods. Thus, we propose that the stretching d* takes the form

4.11 $$d^{*}(x)=x\frac{\alpha }{m-1}\frac{2nD}{L-\Delta }+d_{0},$$

for some constant *α*. Since *d*_0_ = 1/(*m* − 1), the factor of 1/(*m* − 1) cancels in the ratio d*/*d*_0_ used in equation (4.3). Hence, working through the same steps as in the above section, we obtain the same critical buckling criterion in equation (4.1), but now we recognize the parameter *α* is additionally accounting for the variation in curvature across the filaments in the bundle.

### 4.7. The constant *α*

**Figure 9.**
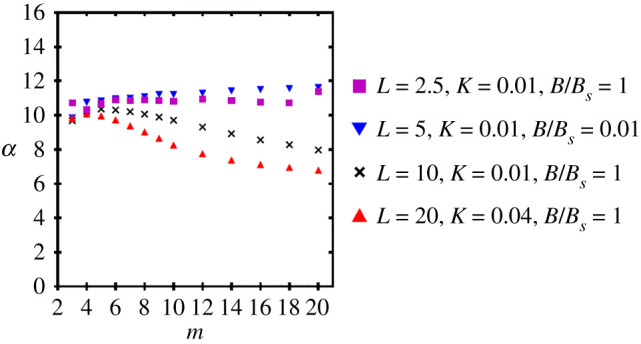
Values of the parameter *α* backed out form numerically obtained internal buckling loads $N_{c}^{*}$ and the formula given by equation (4.1). These correspond to different values of the parameters *B*^in^, *K*, *L*. For each set, we systematically vary *m*.

For the results in figure 8*b*, a value of *α* = 10.61 provides an excellent fit to the data across a wide variety of parameter sets (*B*^in^, *K*, *L*) for a bundle with *m* = 10. Our simple assumption in equation (4.1) is that *α* is independent of all the system parameters. We now further test the variation in the value of *α* with the bundle number *m*. To do so we locate the critical buckling $N_{c}^{*}$ numerically by steadily increasing *N*_*c*_ until a perturbation about the undistorted bundle state is found to develop internal buckling. We then use the predicted internal buckling formula in equation (4.1) to calculate the value of *α* for each set (*K*, *B*^in^, *L*, *m*). The results for a range of parameters are shown in figure 9. For lower length filaments the parameter is approximately constant with *m*, whereas for the longer filaments there is some weak decay with *m*. This implies that the simple first-order model misses some of the more subtle aspects of the general nonlinear model. For instance in the general model, the deformation shape is more complex relative to the triangular approximation, the deformation magnitude is suppressed at either end of the filament by the boundary conditions, the end displacement at the unpinned end is not uniform, and the outer rods may deform slightly. However, all values of *α* are of the same order of magnitude as the value of 8 in equation (4.10), indicating the model is predicting the correct scaling, resulting from balancing the elastic and bending energies.

### 4.8. A note on confinement and wavemode

It is well known that filaments in embedded media buckle at higher wavemodes than the free filament model (see [7–9,41]), with the mode determined by the stiffness of the embedding/constraining medium. In this case, the stiff outer filaments/sheath act to confine the filaments. The formula for the critical mode n* in equation (4.10) indicates that the spring stiffness *K* and bundle length to width ratio *L* promote higher mode buckling. The confinement means that the peak buckling amplitude experienced by the central filaments must decay in the filaments to the outer edge of the bundle and this differential deformation leads to stretching of the spring medium, which, as discussed above, is lower for high wavemodes. Increasing *K* means the benefit of adopting high wavemodes and decreasing this differential stretching is increased, thus promoting higher modes. One can also see from equation (4.3) that increasing the length *L* leads to an increase in the elastic energy for a given mode *n*, thus the energy can be lowered by increasing the mode *n*. Once again this energy is only important because of the confinement leading to differential deformation of the filaments and hence spring stretching. By contrast, it is unsurprising that increasing the stiffness of the filaments *B*^in^ promotes lower wavemodes, a factor not directly related to the confinement.

## 5. Conclusion

We have developed a constitutively simple (in terms of its material parameters) model for interactive elastic filament bundles via an energy functional, equation (2.2), which accounts for: the filament bending, the filament interaction and non-overlapping constraints, bundle loading and boundary conditions. This minimal model exhibits a diverse range of free energy minima, which we categorized into four distinct classes: unbuckled, globally buckled, pinched and internally buckled. Importantly, these follow many biologically observed buckling phenomena, such as those shown in figure 1. These numerical experiments were shown to mimic biologically observed buckling responses.

The model is detailed in §2. A key feature we highlighted, to ensure numerical and physical reliability, was the non-local overlapping potential. This was critical to mediate the complex nonlinear interaction inherent to the model. We believe this insight could be of significant benefit to researchers developing similar theoretical models.

A common feature of many biological filament bundles is the embedding of the system in a stiff matrix or sheath. Interestingly, for sufficiently stiff outer sheaths, the buckling mode was observed to change dramatically from a Euler-like global buckling to an internal buckling mode. We derived both the scaling and analytical formula to predict the critical compressive load leading to this internal buckling transition. As internal buckling leads to large, highly localized pressures (relative to a simple pinching mode), we predict internal collapse may describe catastrophic mechanical failure in biological systems.

Furthermore, the derived critical compressive load, given in equation (4.1), is remarkably straightforward. We highlight that the relevant parameters of this prediction should be relatively uncomplicated to estimate. The bending stiffness *B*^in^ can be derived from the filament’s Young’s modulus and Poisson ratio, while the spring/matrix stiffness *K* is a measure of the resistance to deformation of the material/bonds connecting the filaments. Thus, we believe it has significant predictive potential for applications of biological filament bundles in supported environments, where internal filament collapse plays a key role.

Possible future investigations using this model might also address what happens if the filaments are of different types (they have variable bending stiffness), or have structural weakness (bending stiffness varying with arclength). One can also consider variations on the pinching force such as pinching at multiple points or the pinching/pulling of individual filaments (plucking). In each case, one would have access to a richer variety of geometries than presented here, but we believe the principle of competition between elastic matrix stretching and filament bending highlighted here would still play a key role in determining these geometries.

The model presented here is two-dimensional, but has relevance to three-dimensional bundles under the condition that there are no torsional stresses. Under such conditions, the pinching modes presented here could represent a cross-section of an axially symmetric bundle pinching with uniformly applied pinching pressure. This would be relevant for the optic nerve where the hoop stress in the scleral wall applies a uniform pressure to the lamina cribosa [4]. The global buckling mode represents a planar buckling of a three-dimensional bundle, this behaviour has been observed in related global buckling models [19]. Some caution can be found in results in a related model of a filament connected to a fixed surface found in [41]. In this case, there is some indication that the spring connection can lead to a competition between non-planar and planar buckling modes for the lowest-load collapse, although both have similar wavelengths. Similar considerations would apply to the internal buckling modes. However, it remains the case that the internal buckling wavelength would still be determined by the balance between the elastic stiffness penalizing higher wavemodes and the interaction force promoting them. This is a critical principle underlying internal buckling modes in such systems.

## Data Availability

This article does not contain any additional data.

## Appendix A. Dimensional unit summary

In table 1, we summarize the parameters used throughout in the filament bundle model and internal buckling model. We also indicate the dimensions of each of these parameters, prior to the non-dimensionalization procedure in §2.7.

**Table 1.** Summary of model parameters with dimensional analysis prior to non-dimensionalization. M, L and T, are mass, length and time units, respectively.

parameter	description	dimensions
*L*	filament length	(L)
*l*	segment length	(L)
*W*	bundle width	(L)
*θ*	segment angle	()
*N*_*p*_	vertical pinching load	(ML T^−2^)
*N*_*c*_	horizontal compressive load	(ML T^−2^)
*d*	spring length	(L)
*A*	triangular area of matrix	(L^2^)
*B*	bending stiffness	(ML^3^ T^−2^)
*K*	spring stiffness	(ML T^−2^)
*d*	vertical point displacement	(L)
Δ	horizontal point displacement	(L)

## Appendix B. Gradient expressions

In this appendix, we detail the gradient expressions to the various terms in our energy functional. They are employed in our L-BGFS minimization algorithm.

We leave the interaction energy until last as it is by far the most complex.

**B.1. Bending energy *E*_*b*_**

The explicit gradient terms for the bending energy are

B 1 $$\begin{array}{cc} & \frac{\partial E_{b}}{\partial \theta_{ij}}=\frac{B_{ij}}{2l}\tan\left(\frac{\theta_{ij}-\theta_{i(j-1)}}{2}\right)\sec^{2}\left(\frac{\theta_{ij}-\theta_{i(j-1)}}{2}\right)\\ \mathrm{and}\; & -\frac{B_{i(j+1)}}{2l}\tan\left(\frac{\theta_{i(j+1)}-\theta_{ij}}{2}\right)\sec^{2}\left(\frac{\theta_{i(j+1)}-\theta_{ij}}{2}\right). \end{array}\}$$

**B.2. Loading potentials *E*_*l*_**

For the loading potentials,

B 2 $$\frac{\partial E_{l}^{1}}{\partial \theta_{ij}}=-N_{ci}l\sin\theta_{ij}$$

and

B 3 $$\frac{\partial E_{l}^{1}}{\partial \theta_{ij}}=\sum_{i=1}^{m}\left[l\cos\theta_{ij}\left(\sum_{k=j}^{m}N_{\;p(ik)}\right)\right].$$

**B.3. Constraint potentials *E*_*c*_**

For the constraint potentials,

B 4 $$\frac{\partial E_{c}^{1}}{\partial \theta_{ij}}=\frac{\partial H}{\partial y_{\mathrm{in}}}\frac{\partial y_{\mathrm{in}}}{\partial \theta_{ij}}=-l\frac{\partial H}{\partial y_{\mathrm{in}}}\sin\theta_{ij}$$

and

B 5 $$\frac{\partial E_{c}^{2}}{\partial \theta_{ij}}=\left\{ \begin{array}{cc} \frac{\partial H}{\partial \theta_{i1}} & \text{if}\;j=1,\\ \frac{\partial H}{\partial \theta_{\mathrm{in}}} & \text{if}\;j=n,\\ 0 & \text{otherwise}. \end{array}\right.$$

**B.4. Interaction potentials *E*_in_**

Note that each angle *θ*_*ij*_ affects not only all *d*_*ik*_ and $d_{ik}^{\pm }$ for *j* ≤ *k* but also all *d*_(*i*−1)*k*_ and $d_{(i-1)k}^{\pm }$ (again for for *j* ≤ *k*). Here,

B 6 $$\frac{\partial E_{\mathrm{in}}^{1}}{\partial \theta_{ij}}\sum_{k=j}^{n}l\frac{\partial \;f}{\partial d_{ik}}\frac{\partial d_{ik}}{\partial \theta_{ij}}+\sum_{k=j}^{n}l\frac{\partial \;f}{\partial d_{(i-1)k}}\frac{\partial d_{(i-1)k}}{\partial \theta_{ij}}.$$

The diagonal spring terms are similar

B 7 $$\frac{\partial E_{\mathrm{in}}^{1+}}{\partial \theta_{ij}}=\sum_{k=j}^{n-1}l\frac{\partial \;f}{\partial d_{ik}^{+}}\frac{\partial d_{ik}^{+}}{\partial \theta_{ij}}+\sum_{k=j-1}^{n-1}l\frac{\partial \;f}{\partial d_{(i-1)k}^{+}}\frac{\partial d_{(i-1)k^{+}}}{\partial \theta_{ij}}.$$

(Note the *i* − 1 spring term starts at *k* = *j* − 1 not *k* = *j* and there is no + diagonal spring at *n*.) Similarly

B 8 $$\frac{\partial E_{\mathrm{in}}^{1-}}{\partial \theta_{ij}}=\sum_{k=j}^{n}l\frac{\partial \;f}{\partial d_{ik}^{-}}\frac{\partial d_{ik}^{-}}{\partial \theta_{ij}}+\sum_{k=j+1}^{n}l\frac{\partial \;f}{\partial d_{(i-1)k}^{-}}\frac{\partial d_{(i-1)k^{+}}}{\partial \theta_{ij}}.$$

These are completed with the following expressions for derivatives of the spring lengths:

$$\begin{array}{rl} & \frac{\partial d_{ik}}{\partial \theta_{ij}}=\left\{ \begin{array}{cc} \frac{l}{d_{ik}}[x_{ik}^{d}\sin\theta_{ij}-y_{ik}^{d}\cos\theta_{ij}] & \text{if}\;k>j+1,\\ 0 & \text{otherwise } \end{array}\right.\\ & \frac{\partial d_{ik}^{\pm }}{\partial \theta_{ij}}=\left\{ \begin{array}{cc} \frac{l}{d_{ik}^{\pm }}[x_{ik}^{d\pm }\sin\theta_{ij}-y_{ik}^{d\pm }\cos\theta_{ij}] & \text{if}\;k>j+1,\\ 0 & \text{otherwise} \end{array}\right.\\ & x_{ik}^{d}=x_{(i+1)k}-x_{ik},\;y_{ik}^{d}=y_{(i+1)k}-y_{ik}\\ \mathrm{and}\; & x_{ik}^{d\pm }=x_{\left(i+1)(k\pm 1\right)}-x_{ik},\;y_{ik}^{d\pm }=y_{\left(i+1)(k\pm 1\right)}-y_{ik}. \end{array}$$

To get the derivatives of *d*_(*i*−1)*k*_, we simply replace (*x*_*i*+1*k*_ − *x*_*ik*_) with −(*x*_*ik*_ − *x*_(*i*−1)*k*_) and similarly for the *y* difference. This same adjustment applies to the derivatives of $d_{(i-1)k}^{\pm }$.

Finally, we calculate the derivatives of the non-overlap potential $E_{\mathrm{in}}^{2}$. Rather than list each derivative we give an example based on the term $A(d_{ij},d_{ij}^{+})$. One term involved in the derivative of this contribution will be

B 9 $$\frac{\partial g(A(d_{ik},d_{ik}^{+}))}{\partial \theta_{ij}}=\frac{\partial g}{\partial A}\frac{\partial A}{\partial x_{ik}}\frac{\partial x_{ik}}{\partial \theta_{ij}},$$

where,

B 10 $$\frac{\partial g}{\partial A}=-\frac{C_{1}C_{2}}{A_{0}}e^{-C_{2}A/A_{0}},$$

B 11 $$\frac{\partial A}{\partial x_{ik}}=-/frac12(y_{\left(i+1)(k+1\right)}-y_{ik}),$$

and

B 12 $$\frac{\partial x_{ik}}{\partial \theta_{ij}}=\left\{ \begin{array}{cc} -\sin\theta_{ij} & \text{if}\;k\geq j.\\ 0 & \text{otherwise}. \end{array}\right.$$

This derivative would feature in all $\partial A(d_{ik},d_{ik}^{+})/\partial \theta_{ij}$ of *k* ≥ *j*. There will also be terms including partial derivatives of *A* with respect to *x*_(*i*+1)*k*_, *x*_(*i*+1)(*k*+1)_, *y*_(*i*+1)*k*_, *y*_*ik*_, and *y*_(*i*+1)(*k*+1)_. The total derivative is then a matter of straightforward if tedious book-keeping by applying this to all $A(d_{ik},d_{ik}^{+})$, $A(d_{ik},d_{ik}^{-})$, $A(d_{i(k+1)},d_{ik}^{+})$ and $A(d_{i(k-1)},d_{ik}^{-})$.
